# Idiosyncratic bone responses to blood flow restriction exercise: new insights and future directions

**DOI:** 10.1152/japplphysiol.00723.2022

**Published:** 2023-11-23

**Authors:** Luke Hughes, Christoph Centner

**Affiliations:** ^1^Department of Sport Exercise & Rehabilitation, Northumbria University, Newcastle upon Tyne, United Kingdom; ^2^Department of Sport and Sport Science, University of Freiburg, Freiburg, Germany; ^3^Praxisklinik Rennbahn, Muttenz, Switzerland

**Keywords:** blood flow restriction, bone remodeling, exercise, hypoxia

## Abstract

Applying blood flow restriction (BFR) during low-load exercise induces beneficial adaptations of the myotendinous and neuromuscular systems. Despite the low mechanical tension, BFR exercise facilitates a localized hypoxic environment and increase in metabolic stress, widely regarded as the primary stimulus for tissue adaptations. First evidence indicates that low-load BFR exercise is effective in promoting an osteogenic response in bone, although this has previously been postulated to adapt primarily during high-impact weight-bearing exercise. Besides studies investigating the acute response of bone biomarkers following BFR exercise, first long-term trials demonstrate beneficial adaptations in bone in both healthy and clinical populations. Despite the increasing number of studies, the physiological mechanisms are largely unknown. Moreover, heterogeneity in methodological approaches such as biomarkers of bone metabolism measured, participant and study characteristics, and time course of measurement renders it difficult to formulate accurate conclusions. Furthermore, incongruity in the methods of BFR application (e.g., cuff pressure) limits the comparability of datasets and thus hinders generalizability of study findings. Appropriate use of biomarkers, effective BFR application, and befitting study design have the potential to progress knowledge on the acute and chronic response of bone to BFR exercise and contribute toward the development of a novel strategy to protect or enhance bone health. Therefore, the purpose of the present synthesis review is to *1*) evaluate current mechanistic evidence; *2*) discuss and offer explanations for similar and contrasting data findings; and *3*) create a methodological framework for future mechanistic and applied research.

## Graphical abstract

**Figure:**
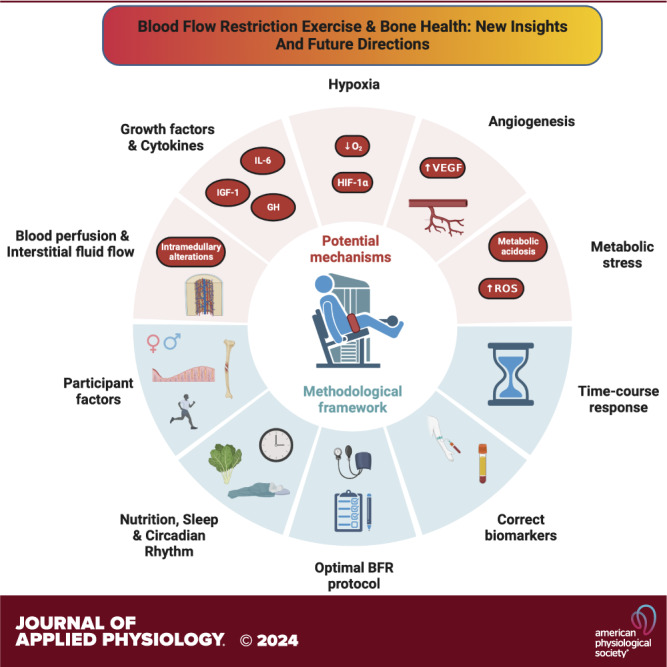

## INTRODUCTION

Prolonged mechanical unloading has repeatedly been demonstrated to result in a significant maladaptation of the skeletal system (1). In conditions of immobilization, weightlessness, or reduced physical activity a lack of mechanical stress on the bone tissue facilitates a cascade of catabolic events ultimately leading to downregulation of bone metabolism and thus influencing bone health. Additionally, changes in hormonal status (2) or aging per se (3) might be important factors, which have been demonstrated to have detrimental effects on the osteogenic milieu. According to Wolff’s law, bone remodeling and trabecula organization are strongly associated with the magnitude and direction of stress and strain (4). This is supported by current recommendations from the American College of Sports Medicine stating that moderate- to high-load resistance exercises or high-impact exercises (e.g., jumping) are necessary to induce optimal adaptations on the bone level and increase bone health (5). Although populations at high risk of microarchitectural deterioration of bone tissue (such as postmenopausal women, frail elderly or immobilized patients) have been demonstrated to greatly benefit from systematic exercise programs (6, 7), disease progression and frequently occurring comorbidities in conjunction with high bone fracture risks might limit them to training regimes with low loading.

In recent years, the combination of blood flow restriction (BFR) during low-load exercise (LL-BFR) at 20–40% of one repetition maximum (1RM) or <40% of maximal aerobic capacity has been demonstrated as a viable alternative for facilitating muscular (8), tendinous (9, 10), and skeletal (11, 12) adaptations. Many such adaptations are comparable in magnitude to those observed as a result of high-load exercise training (13, 14). By partially and completely restricting arterial inflow and venous outflow, respectively, within the working limb, the resultant ischemia, hypoxia, and metabolic stress have been shown to trigger a cascade of physiological processes involved in tissue adaptation (15). Interestingly, recent evidence revealed that this training methodology is effective in promoting an osteogenic response of the bone material, although this has previously been postulated to adapt primarily during high-impact weight-bearing exercise (5).

With respect to the current BFR literature investigating the impact on bone metabolism and health, there is substantial heterogeneity regarding study design, selected biomarkers, participant characteristics, and time course of measurement, which limits the formulation of accurate conclusions. First, previously used BFR protocols greatly differ regarding BFR pressure intensity (i.e., arbitrary vs. individualized pressures) and duration and muscle groups exercised. Second, several key biomarkers should be assessed within a holistic approach to thoroughly investigate the bone remodeling response to exercise and thus identify the physiological mechanisms. Third, limited data exist that have investigated the time course of the osteogenic response following LL-BFR exercise. Therefore, the overall aims of the present review are to *1*) evaluate current mechanistic evidence; *2*) discuss and offer explanations for similar and contrasting data findings; *3*) discuss the potential mechanisms by which LL-BFR exercise may trigger an osteogenic response; and *4*) create a methodological framework for future mechanistic and applied research.

## MECHANISMS OF BONE REMODELING

### Bone Remodeling Process

Bone remodeling is a fundamental process by which skeletal tissue is constantly renewed to maintain bone strength and mineral homeostasis, thus preserving its essential load bearing and protective functions (16). Remodeling of skeletal tissue throughout the life span is critical for repair of microdamaged bone and removal of old bone and replacing it with new, mechanically stronger bone in response to daily physical load, changing biomechanical forces, and aging effects. It is an active and dynamic process that relies on the correct balance and tight coupling between osteoclastic bone resorption and osteoblastic bone formation (17) and can occur in a targeted and a nontargeted manner. The process is primarily orchestrated by osteocytes (18), which are terminally differentiated osteoblasts found within the bone matrix and account for >90% of adult bone cells (19). These cells act as the skeletal mechanosensors, responding to microdamage and changes in loading by initiating bone remodeling and limiting bone resorption and formation processes to preserve bone mass. Additionally, they respond to hormones, e.g., parathyroid hormone (PTH), to initiate bone resorption and thus maintain mineral homeostasis (18).

**Figure 1.**
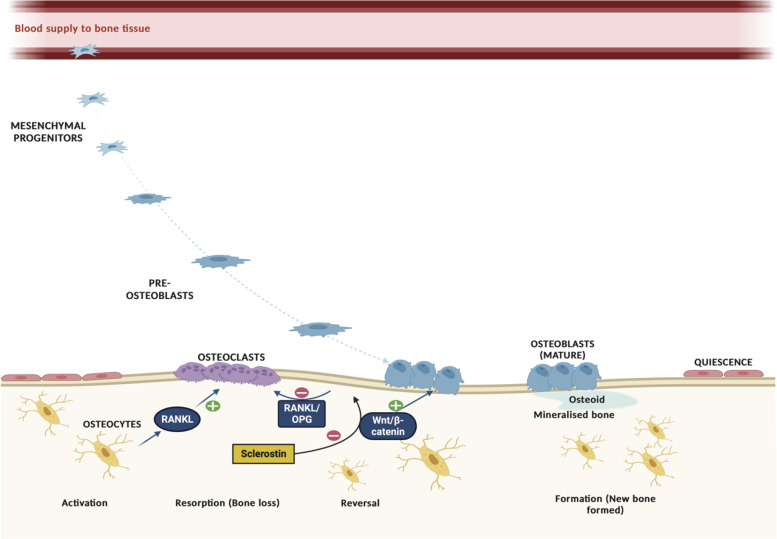
Overview of the bone remodeling process. Osteocytes initiate bone remodeling and limit bone resorption and formation processes to preserve bone mass. The RANKL/RANK/OPG and Wnt/β-catenin pathways are 2 key signaling pathways that regulate the differentiation and function of osteoclasts and osteoblasts. Osteocytes stimulate osteoclastogenesis via production of RANKL at the initiation of the bone remodeling cycle. RANKL then binds to osteoclastic precursor cells, stimulating osteoclast differentiation and facilitating fusion. Osteoblasts and osteocytes secrete OPG, which binds to RANKL, inhibiting osteoclastic bone resorption by preventing its binding to RANK. The Wnt/β-catenin pathway facilitates osteoblast differentiation. Sclerostin reduces osteoblastic bone formation by inhibiting canonical Wnt/β-catenin signaling. Created with BioRender with permission.

In response to direct endocrine activation or osteocyte-derived signals, osteoblasts recruit osteoclast precursors to the remodeling site for resorption of damaged/old bone. After resorption, a phase of formation begins with replacement of osteoclastic cells with osteoblastic cells. In this phase, osteoblasts synthesize and secrete a type I collagen-rich osteoid matrix and bone mineralization (i.e., new bone formation) occurs. Osteocytes play a key role in signaling the end of remodeling by secretion of antagonists to osteogenesis (20). Upon completion of mineralization, osteoblasts undergo apoptosis or terminally differentiate into osteocytes and become entombed within the bone matrix (18), and the resting bone surface environment is maintained until the next wave of remodeling. Figure 1 presents an overview of the bone remodeling process.

### Signaling Pathways

Targeted bone remodeling occurring simultaneously at anatomically distinct sites indicates that local regulation is critical to balance the processes of bone resorption and formation. Two key signaling pathways are responsible for transducing locally and systemically produced signals: *1*) RANKL/RANK/OPG and *2*) Wnt/β-catenin (18). Together these pathways regulate osteoclast and osteoblast differentiation and function, and they also serve as the mechanism by which several hormones exert their actions on the bone remodeling process (18). Within the RANKL/RANK/OPG pathway, it is predominantly osteocytes within the bone matrix that sense changes in load and microdamage and stimulate osteoclastogenesis via production of RANKL at the initiation of the bone remodeling cycle (21, 22). When RANKL then binds to its receptor RANK on osteoclastic precursor cells, it stimulates osteoclast differentiation and facilitates fusion, activation, and survival (18, 23). Furthermore, RANKL-RANK binding induces downstream signaling molecules including tumor necrosis factor-receptor associated factor 6 and mitogen-activated protein kinase and ultimately activates key transcription factors that regulate osteoclast gene expression (18, 24, 25). Conversely, osteoblasts and osteocytes secrete OPG, a decoy receptor for RANKL, which can inhibit osteoclastic bone resorption by binding to RANKL and preventing its binding to RANK (18, 21). Therefore, the RANKL-to-OPG ratio is key in the bone remodeling cycle (21, 22). The canonical, β-catenin-dependent Wnt signaling pathway is a major regulator of osteoblastic bone formation (18). In the presence of Wnt, Frizzled binds to its coreceptors LPL5/6 and facilitates accumulation of β-catenin in the cytoplasm, which translocates to the cell nucleus and triggers and upregulates genes involved in osteoblast differentiation (26). For a detailed overview of the RANKL/RANK/OPG and Wnt/β-catenin pathways, the reader is directed to previous reviews (22, 26).

### Endocrine Mediation

**Figure 2.**
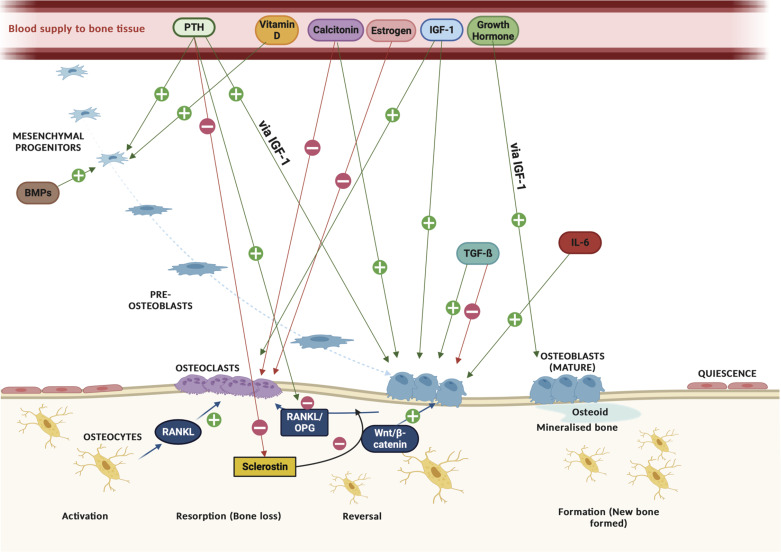
Hormonal and growth factors involved in the regulation of bone remodeling. Parathyroid hormone (PTH) stimulates proliferation and differentiation of mesenchymal progenitors to mature osteoblasts via insulin-like growth factor-I (IGF-I). PTH also induces differentiation of committed mesenchymal progenitors and induces receptor activator of nuclear factor kappa ligand (RANKL) from mature osteoblasts to promote osteoclastogenesis. Vitamin D_3_ stimulates osteoblastogenesis via differentiation of mesenchymal stem cells into osteoblasts. Calcitonin inhibits osteoclast activity to suppress increased bone resorption drive by PTH and also increases osteoblast proliferation and differentiation. Estrogen primarily attenuates osteoclastogenesis by stimulating osteoclast apoptosis. Growth hormone stimulates osteoblast proliferation directly and indirectly via increased IGF-I production. IGF-I stimulates osteoblast proliferation, function, and survival and promotes osteoclast differentiation. Bone morphogenetic proteins (BMPs) induce differentiation of mesenchymal cells to osteoblasts and also enhance the differentiated function of the osteoblast. Transforming growth factor-beta (TGF-β) can both stimulate early osteoblast differentiation and inhibit late osteoblast differentiation. IL-6 induces RANKL production in osteoblastic cells, which in turn stimulates differentiation of osteoclast precursors into mature osteoclasts. Created with BioRender with permission.

The organized expression and release of several hormones and growth factors regulates the bone remodeling process in a systemic and local fashion (Fig. 2). Major hormonal regulators of this process include PTH, calcitonin, vitamin D_3_, estrogen, and growth hormone. The requirement to control blood calcium level is the primary driver behind secretion of PTH (16). PTH is considered a key regulator as it can elicit directly opposing effects on bone remodeling, depending on duration of exposure (18). The main function of PTH is to maintain blood calcium homeostasis via stimulation of bone resorption (27). However, prolonged exposure to continuous PTH (e.g., in individuals with hyperparathyroidism) results in hypercalcemia and bone loss (18) due to modulation of the RANKL/RANK/OPG signaling pathway. On the other hand, intermittent stimulation of the PTH receptor enhances bone formation via modulation of Wnt signaling leading to increased osteoblastogenesis, target gene expression, and enhanced bone formation (18, 28, 29). Moreover, PTH stimulates the proliferation and differentiation of osteoprogenitors to osteoblasts via insulin-like growth factor-I (IGF-I) and along with IGF-I promotes osteoclastogenesis (18). Calcitonin primarily acts to suppress the increased bone resorption driven by PTH by inhibiting the activity of osteoclasts (30), thus complementing the function of PTH, and may also play a role in increasing osteoblast proliferation and differentiation (16). Vitamin D_3_ stimulates osteoblastogenesis via differentiation of mesenchymal stem cells into osteoblasts (31), and depletion of blood vitamin D_3_ levels has been found to induce RANKL-mediated osteoclastogenesis and bone loss (32). Similarly to calcitonin, estrogen primarily attenuates osteoclastogenesis by stimulating osteoclast apoptosis (33). Amassing evidence also highlights the role of growth hormone in bone health. It has been reported that growth hormone stimulates osteoblast proliferation directly (34) and/or indirectly by increasing IGF-I production (35). In both human and animal models, supplementation of growth hormone has been shown to elicit positive effects on bone health such as increases in bone mineral density (BMD) and mass and positive effects on bone turnover indicated by a concurrent increase and decrease of bone formation and bone resorption biomarkers, respectively (36–38). In addition to systemic hormonal regulation, amassing evidence indicates that several growth factors and cytokines play significant roles in the regulation of bone remodeling (16). Figure 2 provides an overview of key factors involved in the regulation of bone remodeling.

### Skeletal Blood Supply

The skeletal and circulatory systems are intricately connected (39), with the skeleton receiving ∼5–10% of resting cardiac output via an extensive network of blood vessels and capillaries (40). The primary blood supply to bone is delivered through one or more nutrient arteries, which penetrate into the medulla and connect to a smaller periosteal arterial supply to perfuse cortical bone (40). The marrow cavity provides several vascular niches that are thought to regulate the growth and differentiation of hematopoietic and stromal cells, partly via gradients in oxygen tension (40). Blood exits the medullary cavity via small veins that penetrate the cortex; therefore perfusion is predominantly centrifugal (40). However, there are differences in the vascular organization of different types of bones, for example, long bones have less periosteal blood supply compared with flat bones (41), and the mechanisms that regulate bone blood flow are not fully understood (42). Nevertheless, blood supply is critical to bone health as it provides oxygen, essential nutrients, and regulatory factors for bone metabolism and removes metabolic waste products, permitting a greater degree of cellularity, remodeling, and repair compared with more avascular tissues such as cartilage (40). Blood supply to the bone plays a critical role in bone growth (42), fracture repair (43), and disease (44), and in the absence of sufficient and well-regulated blood flow, bone cannot maintain its integrity (45). Indeed, chronic insufficient blood supply to the bone (e.g., in peripheral arterial disease) is associated with bone loss (46), impaired growth (46), delayed fracture healing (47), and increased fracture risk (48).

Several intrinsic and extrinsic factors can affect blood supply to the bone both acutely and chronically. From an acute perspective, exercise loading and vasoconstriction of blood vessels can affect blood supply to the bone (45, 49), similarly to the muscles. Exposure to microgravity and ground-based analogs (i.e., head-down tilt), which reduces axial loading, can affect macro- and microvascular blood supply to the tibia bone (50, 51). Astronauts returning from long-duration missions on the International Space Station present with loss of bone mineral density (52, 53), and it is thought that this may be a consequence of chronically altered bone perfusion due to cephalad fluid shifts that occur in the absence of axial loading (54). Such evidence has led many to explore the possible role of impaired bone hemodynamics in triggering pathological states. A key factor affecting bone blood flow (and overall bone health) is age. The composition and mechanical properties of bone vary as a function of age (55), and data suggest that bone marrow blood perfusion and reperfusion (i.e., after an ischemic event) decrease with age (56, 57). Aging is associated with enhanced adipogenesis and adipocyte accumulation in bone marrow cavities (58, 59). As osteocytes and adipocytes share a common precursor cell in the bone marrow (i.e., bone marrow stromal cells), it is widely thought that decreased bone formation observed during aging is the result of enhanced adipogenesis versus osteoblastogenesis from these bone marrow precursor cells, essentially impairing osteogenic and hematopoietic regeneration (59, 60). Furthermore, accumulation of adipose tissue in bone marrow increases significantly with osteoporosis (58, 61), and it is thought that vascular degeneration associated with aging and arteriosclerosis may further alter blood perfusion in bone tissue, possibly resulting in ischemia (62, 63).

## EXERCISE AND BONE REMODELING

In populations characterized by bone loss or increased susceptibility to fractures (i.e., astronauts and osteoporosis), exercise is an effective preventive and treatment strategy (64, 65). It is well supported that the primary stimulus for bone anabolism is physical loading (66), alongside several metabolic signals including altered pH, production of reactive oxygen species (ROS), and availability of blood calcium (67–69). Existing evidence indicates that bone responds to the mechanical and metabolic stimuli of exercise predominantly through remodeling (70). Importantly, as bone responds to the magnitude, frequency, and direction of exercise-induced loading cycles (70, 71) different forms of exercise exert activity-specific mechanotransducive signals and loading patterns (72).

Several acute and chronic measures of bone can be used to study the effects of exercise. Chronic indicators of intervention effectiveness include measurement of bone microarchitecture (assessed by high-resolution peripheral quantitative computed tomography) and/or BMD (assessed by dual-energy X-ray absorptiometry). However, as highlighted by Dolan et al. (71) bone responds slowly to stimuli and measurable changes can take several months to years to occur; therefore these assessment techniques cannot be used to detect the acute response of bone to exercise. Importantly, bone (re)modeling markers can be used to assess dynamic bone activity and the acute response to exercise. These markers are products of bone proteins and/or cells that are present in the systemic circulation and exemplify the processes of bone formation and resorption (71). To accurately examine the mechanisms by which bone responds to mechanical loading and metabolic stimuli, it is important that biomarkers measured are specific to bone tissue and demonstrate small biological variability to enable accurate mechanistic interpretation. The National Bone Health Alliance recommends that, as a minimum, studies investigating bone metabolism must include NH_2_-terminal propeptide of type 1 procollagen (P1NP) and COOH-terminal telopeptide of type I collagen (CTX-1) as biomarkers of bone resorption and bone formation, respectively, as these markers demonstrate higher specificity to bone metabolism and smaller biological variability compared with other markers (73). Furthermore, PTH and calcitonin measurements serve as useful biomarkers of calcium metabolism. Below in this section, the bone metabolic response to acute and chronic exercise is briefly discussed, in addition to factors that influence these processes and the types of training for improving bone health. For detailed reviews and meta-analyses, the reader is directed to the work of Dolan et al. (71, 74).

### Effects of Acute Exercise on Bone Remodeling and Mediating Factors

Previous scientific evidence in the field of bone remodeling following acute exercise indicates that an acute bout of exercise typically elicits a pronounced increase in bone resorption (71, 75–78). This increase in bone resorption following mechanical loading is largely driven by a change in CTX-1 (74), with small to moderate effects observed within 15 min and up to 2 h after exercise (74). Circulating levels of CTX-1 typically return to baseline >2 h after exercise; however, some studies have reported elevated levels of CTX-1 at 72 h after exercise. Several exercise-induced metabolic changes such as a reduction in serum calcium and subsequent secretion of PTH, which peaks within the first 15 min after cessation of exercise (74), may stimulate osteoclast activation and thus bone resorption. Although some studies have reported increased bone formation following acute exercise, evidence regarding changes in bone formation is still conflicting, with some studies showing increases in bone formation (COOH-terminal propeptide of type I collagen) for a period of 4–6 h after exercise (79) whereas others found only small effects (71, 74). The systematic review by Dolan et al. (74) suggests minimal anabolic bone responses measured by P1NP in response to acute exercise. However, this study pooled outcomes across all designs and categories and thus likely neglected to control for several key influencing factors when investigating bone remodeling process in response to exercise, including nutrient intake and timing, circadian timing, intervals between acute exercise bouts, and the momentum and impulse of the exercise stimulus, which are discussed in more detail throughout. It is important to note that acute exercise can produce a several-hours-long increase in P1NP, providing these factors are accounted for. It is evident that variability in outcomes might be caused by time-specific and transient response of different biomarkers to acute exercise bouts (71, 74) (Table 1).

**Table 1.** Time course response of key biomarkers of bone metabolism

Biomarker	Time Points	Rationale
CTX-1	Within 15 min and up to 2 h after exercise. Some increase at 72 h after exercise	Key biomarker of bone resorption
P1NP	Within 15 min after exercise and for up to 4–6 h after exercise	Key biomarker of bone formation
PTH	Peak immediately after exercise. Return to baseline within 15 min	Useful biomarker of calcium metabolism

CTX-1, COOH-terminal telopeptide of type I collagen; P1NP, NH_2_-terminal propeptide of type 1 procollagen; PTH, parathyroid hormone.

An important factor that needs to be considered when prescribing exercise with the aim of inducing biopositive bone remodeling is exercise timing and frequency. Previous animal studies indicate that there is a decline in mechanosensitivity of bone cells dependent on recovery time after exercise (80, 81). The findings suggest a mechano-refractoriness immediately following a single bout of mechanical loading with restoration of mechanosensitivity after 6–8 h (81). Comparable effects have also been found in humans. Besides frequency of mechanical loading, exercise impulse (82) and circadian timing are also thought to play a key role in mediating the magnitude of bone remodeling. Circadian timing is primarily related to synergistic anabolic effects of exercise and several bone biomarkers operating in a circadian fashion (e.g., PTH or CTX) (83–85). In this regard, previous studies indicated that PTH administration (via injection) in osteoporotic postmenopausal women at 8 AM abolished the nocturnal CTX peak, whereas the same injection at 8 PM allowed the full circadian CTX secretion (86). Given that exercise reportedly increases PTH secretion, it might be assumed that exercise timing (in the morning) might be an essential factor for eliciting long-term adaptations (87). Exercise impulse is the duration of an effective exercise stimulus necessary to produce a biological, anabolic effect. With regard to bone, previous data suggest that the effect of exercise on bone remodeling depends on its impulse at an appropriate exercise intensity, with the required impulse being a minimum of 40–45 min for walking exercise, for example (82). Moreover, the relationship between exercise and biomarker response is further mediated by nutrient intake (79, 88). The results from a recent clinical trial in diabetic postmenopausal women showed that the exercise-induced anabolic response on the bone is dependent on nutrient intake before exercise. Findings from Borer and colleagues (79) indicated that only meals before exercise induced anabolic responses, whereas meals following the exercise did not, providing a sufficient exercise impulse was provided within circadian constraints. The importance of sufficient nutrients was also confirmed within a recent animal model (89). The findings showed that reintroduction of food after a 16-h fast markedly increased new cortical bone in tibia and further increased the response of the bone to mechanical stimuli. Interestingly, comparable to the mechano-refractoriness mentioned above, studies suggest that the restoration of bone nutrient sensitivity to exercise also follows a 5- to 8-h rhythm (82). These factors that are known to influence the bone remodeling process must be accounted for and, where necessary, controlled for in future studies.

### Effects of Chronic Exercise on Bone Remodeling

In contrast, an increase in resting levels of bone formation biomarkers, mainly P1NP and bone-specific alkaline phosphatase (ALP), is consistently observed with chronic exercise training interventions, whereas most studies report no change or even a reduction in resting level of bone resorption biomarkers with chronic training (71). Together, these changes in resting biomarkers suggest that chronic exercise training upregulates bone formation processes and leads to a bone metabolic profile favoring formation. Several studies have observed an increase in BMD concomitant with increases in biomarkers of bone formation (90–92), demonstrating how biomarkers of remodeling can be used to monitor the effectiveness of chronic training. However, it is important to note that biomarkers of bone metabolism are systemic and cannot identify bone activity at any specific site, which is problematic because the true bone response to loading is largely site specific (93). Importantly, several factors are known to influence the bone metabolic response to exercise, and certain biomarkers are more susceptible to these confounding effects than others (74). For example, CTX-1 is known to be influenced by circadian rhythm and nutritional status to a greater extent than P1NP (71, 94). Other potential confounding factors include age, sex, nutritional status and energy availability, circadian variations, menstrual cycle, injury history, and various medical conditions (71, 95–98). Although the purpose of this review is not to discuss these potential confounding factors in great depth, this highlights the importance of standardization of specific conditions and overall study design, to ensure that valid data are obtained from which to draw accurate conclusions (71).

## BFR TRAINING AND BONE REMODELING

At present, there is a limited understanding of the primary and optimal stimuli for bone anabolism; however, the existing data suggest that exercise involving higher impact loads and/or longer durations that reflect, arguably, greater metabolic demand elicits an acute response in bone tissue. Interestingly, research during the last decades has reported that using a low mechanical loading (LL) combined with blood flow restriction (BFR) elicits anabolic responses on the muscular (8) and tendinous (10) levels and also effects bone remodeling (99). Although the available literature regarding the effects of LL-BFR training on bone metabolism is still scarce, both acute and long-term trials suggest that this type of exercise may elicit positive effects on bone. The following subsections discuss available evidence on the acute and chronic effects of LL-BFR training on bone metabolism and health.

### Acute Effects

In young men, Bemben et al. (100) investigated the effects of a single bout of LL-BFR exercise (20% 1RM) for the knee extensors and flexors on bone formation and resorption markers. The results revealed that LL-BFR exercise resulted in a significant reduction of bone resorption markers (i.e., CTX-1) compared with low-intensity exercise alone. Within a recent follow-up experiment (101) the same research group compared high-load exercise (70% 1RM) and moderate-load exercise (45% 1RM) with LL-BFR exercise (20% 1RM). Interestingly, the authors reported comparable changes in bone formation markers (i.e., bone ALP) between high-load exercise training and LL-BFR exercise training, with no changes in the group with moderate loading (101). However, in this study no between-group differences in bone resorption markers (i.e., CTX-1) were found. In a recent experiment by Copatti and colleagues (102), the authors investigated the effect of different BFR cuff pressure intensities on the magnitude of changes in bone resorption (i.e., PTH) and formation (i.e., bone ALP) markers. In a crossover design, 12 women completed low-load resistance exercise (30% 1RM) without BFR and with BFR applied at 70% and 130% of systolic blood pressure. Interestingly, the results highlighted that PTH concentrations were significantly decreased compared with baseline in all groups immediately after exercise but significantly increased at 15 min after exercise only for the BFR conditions. No changes were seen for bone ALP concentrations, and no pressure-dependent change was observed.

### Chronic Effects

The long-term effects of LL-BFR training on bone remodeling have been investigated in numerous studies with different populations. In healthy individuals, Beekley et al. (103) were the first to investigate the chronic effects of LL-BFR training on bone ALP concentrations after a 3-wk training period. Using a longitudinal design, the authors found that the combination of walking with BFR facilitated significant increases in bone ALP concentrations (+10.8%) compared with the same exercise regimen without BFR (change: +0.3%). The extent to which these chronic increases in resting bone ALP concentrations are predictive of changes in BMD was, however, not investigated. Kim et al. (104) compared the effects of 3 wk of LL-BFR training (20% 1RM), high-load training (80% 1RM), and BFR applied without exercise on bone formation (bone ALP) and resorption markers (CTX-1). The findings indicated that although no differences were found in CTX-1 levels between groups, high-load training elicited a significantly greater increase in bone ALP concentrations compared with LL-BFR training or BFR alone.

In healthy older men, Karabulut et al. (105) investigated the effects of 6 wk of LL-BFR and high-load (HL) training on bone formation (bone ALP) and resorption (CTX-1) markers. The authors found that both high-load and LL-BFR training facilitated comparable increases in bone ALP and bone ALP-to-CTX-1 ratio after 6 wk of training. These results might indicate an increase in bone turnover in both exercise groups and suggest that mechanical loading might not be the sole driver of exercise-induced bone remodeling. Although progressive aging is often associated with a decrease in bone health (106), adequate exercise interventions are vital for patients suffering from pathological deteriorations in bone health. In a very recent trial, Linero and Choi (107) combined the assessments of bone mineral density via dual-energy X-ray absorptiometry and bone turnover markers in postmenopausal women with osteoporosis or osteopenia. Participants were randomly assigned to moderate- to high-load training (60% 1RM), LL-BFR training (30% 1RM), low-load training (30% 1RM), or a nonexercising control group. After 12 wk of training, significant increases in bone formation (i.e., P1NP) and resorption (i.e., CTX-1) markers were observed in the moderate- to high-load training group. Importantly, whereas low-load training did not demonstrate any significant changes in bone turnover markers, a significant increase in P1NP was observed with LL-BFR training (107). However, these shifts in biomarker concentrations did not translate into changes in BMD in any of the exercise groups.

A recent pioneering clinical trial by Jack and colleagues (99) reported that 12 wk of LL-BFR training mitigated the loss of bone mineral density and lean mass in patients after anterior cruciate ligament reconstruction, in comparison to the same exercise intervention without BFR. Comparable effects on bone remodeling have been observed with chronic LL-BFR training in both healthy (103) and clinical (107) populations (e.g., osteoporosis). From a mechanistic point of view, previous data indicate that a single bout of LL-BFR exercise can acutely increase biomarkers of bone tissue formation and decrease biomarkers of bone resorption, indicating a positive impact on bone tissue metabolism (100, 101). Collectively, this evidence is compelling considering that although exercise that is high impact and multidirectional and uses high/unaccustomed loads is widely accepted as the optimal osteogenic stimulus (74), LL-BFR exercise is inherently the opposite of these characteristics. The notion of performing LL-BFR exercise to improve skeletal health is even more appealing when considering that exercise with lower impact and repetitive, lower loading cycles does not typically elicit skeletal benefits (108). If BFR can improve skeletal health with low-load exercise, this would have important implications for load-compromised populations, e.g., those with osteoporosis, those with fracture injuries, and older adults.

### Mechanisms

**Figure 3.**
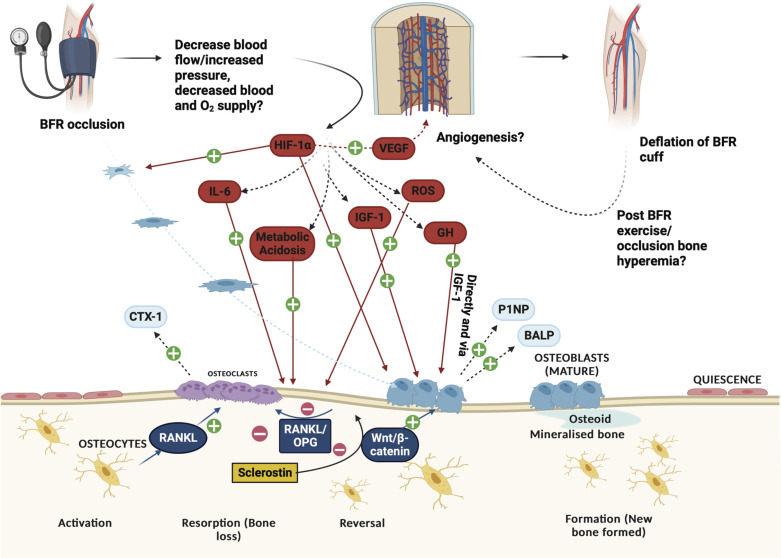
Hypothetical mechanisms of an effect of blood flow restriction (BFR) on bone remodeling processes. Partial or full occlusion of limb blood flow may decrease bone arterial blood perfusion in different areas of bone, causing hypoxia and withdrawal of nutrients. This may lead to a cascade of events including upregulation of several systemic hormones, transforming factors, and cytokines that impact bone remodeling processes. Deflation of the BFR cuff may trigger postexercise bone hyperemia, increasing blood flow to bone tissue. GH, growth hormone; HIF-1α, hypoxia-inducible factor 1-alpha; IGF-1, insulin-like growth factor-I; IL-6, interleukin-6; ROS, reactive oxygen species; VEGF, vascular endothelial growth factor. Created with BioRender with permission.

Despite promising evidence of recent clinical trials that demonstrated beneficial effects of LL-BFR training on acute and chronic bone formation, the underlying mechanisms as well as causal relationships are not well understood. Generally, there are several potential candidates that are speculated to be involved in the osteogenic response seen following training under local hypoxia. These potential mechanisms are discussed below and outlined in Fig. 3.

#### Blood perfusion and interstitial fluid flow.

Mechanical loading during exercise has long been regarded as an essential stimulus for beneficial bone adaptations. Research in previous years has made important steps toward the understanding of how the skeletal system senses stress and strain. In both in vitro and in vivo models, bone interstitial fluid flow has been hypothesized to be a key element for converting and translating mechanical signals into electrical or chemical responses (109). According to the hypothesis, compressive loading (e.g., by exercise) facilitates the development of a pressure gradient leading to increased fluid flow in the lacunar-canicular system from regions with compression to regions with tension (109). Ultimately, the augmentation of fluid sheer stress is speculated to influence bone metabolism by releasing osteogenic signaling factors (110, 111). Because of technical difficulties in directly measuring in vivo interstitial fluid flow, previous studies assessed intramedullary pressure as a marker for intraosseous pressure (112). Although mechanical loading and unloading have been demonstrated to increase and decrease intramedullary pressure (113), respectively, recent experiments have pointed toward the influence of vascular occlusion on changes in intramedullary pressure and interstitial fluid flow. Indeed, Kelly and colleagues (114) found that venous occlusion within their animal model significantly increased intramedullary pressure of the tibia and led to chronic improvements in periosteal new bone formation compared with a control leg. Similar results have been found by further animal experiments (115). Although there is currently scarce evidence regarding these phenomena in humans, it might be speculated that exercise with partial vascular occlusion (as with LL-BFR training) might be beneficial in inducing comparable changes on the bone level (111).

It is currently unknown how LL-BFR exercise per se affects bone blood supply and perfusion, with no published evidence to date. There are some published data that have measured changes in bone blood perfusion with different levels of external pressure, where positive external pressures are similar to the concept of LL-BFR exercise. Mateus and Hargens (39) were the first to investigate the bone hemodynamic response to external pressures in the tibia. In this study, the authors demonstrated how moderate positive pressures up to +30 mmHg, assessed via photoplethysmography, increased bone perfusion in the tibia via the myogenic effect (39). This may occur as a downstream consequence of the myogenic effect in the muscle, with the change at the level of the bone being a secondary myogenic response (39). Interestingly, the authors found that pressures > 30 mmHg (i.e., 50 mmHg in this particular study) decreased bone perfusion, which is likely due to activation of intramuscular pressure receptors, which have an activation threshold of ∼30 mmHg (116). Therefore, it would be reasonable to hypothesize that greater applied pressures during BFR exercise (i.e., 80% of limb occlusion pressure) would result in a greater decrease in bone tissue perfusion during exercise. More recently, Draghici and Taylor (45) reported that with isometric exercise as a sympathoexcitatory stimuli (which would trigger occlusion of vasculature), tibial bone vasculature response was smaller, delayed, and more variable than whole leg vasculature response. These concepts highlight the importance of future studies investigating the effect of LL-BFR exercise on bone blood supply to determine how performing BFR during low-intensity exercise may affect bone perfusion, and whether the magnitude of the applied pressure influences the response. Moreover, given that exercise intensity and load can influence bone blood perfusion, it would be prudent to investigate whether there is an interplay between BFR exercise pressure, load, and volume.

Another potentially key unknown factor of LL-BFR exercise is how deflation of the tourniquet cuff upon completion of exercise may affect bone blood perfusion. There is some evidence that suggests that postexercise bone hyperemia may be correlated with the intensity of muscle contraction (49), which may indicate more forceful contractions causing greater occlusion of arterial blood supply to the muscle and bone tissues and subsequently a greater hyperemia effect. However, data published by Neuschwander et al. (117) suggest that postexercise hyperemia may preferentially increase muscle microvascular flow at the expense of bone microvascular flow, which may indicate shunting of blood to hypoxic muscle tissue. Considering this and the notion that vascular organization can differ across different types of bones with differences in blood supply (e.g., intramedullary blood flow vs. periosteal blood flow), LL-BFR may differentially affect nutrient artery blood flow, intramedullary blood flow, and periosteal blood flow in different bone types. Therefore, future studies should investigate this in tightly controlled experiments. Methods to measure bone blood flow are typically expensive and invasive and are mostly limited to single-time point measurements of bone blood flow (i.e., magnetic resonance imaging, positron emission tomography, and laser Doppler flowmetry) (45), which limits their application in LL-BFR exercise studies. More recently, optical-based technologies such as near-infrared spectroscopy systems have been developed as a potential method for assessing bone blood perfusion noninvasively and continuously (45, 118). In addition, consideration should be given to how factors affecting blood flow to and within the bone may influence the acute and chronic effects of LL-BFR exercise on the bone. For example, because of age-associated adipogenesis (58, 59) and the potential impact on blood perfusion in bone tissue (62, 63), LL-BFR may differentially affect bone blood supply and perfusion in older adults compared with younger individuals, particularly as bone marrow blood perfusion and reperfusion decrease with age (56, 57), which may be due to/further impacted by vascular degeneration with aging.

#### Hormonal and growth factor regulation.

Within the framework of mechanistical explanations of bone metabolism, growth factors have repeatedly been demonstrated to play a vital role. Two of the most investigated growth factors are growth hormone and IGF-I (119) (Fig. 3). Previous studies have shown that growth hormone stimulates osteoblast proliferation directly as well as via IGF-I resulting in increased bone formation (119). Similarly, the absence of growth hormone caused a reduced rate of bone remodeling. Indeed, numerous studies within the BFR literature have reported an increased secretion of growth hormone (120, 121) and IGF-I (122) when training with LL-BFR compared to low-load training alone. For example, Takarada et al. (120) demonstrated that growth hormone significantly increased from before to after LL-BFR exercise, whereas no changes were seen within the control condition with the same exercise but without BFR. However, it must be mentioned that to date there is no evidence regarding the direct mediating effects of growth hormone on bone remodeling following LL-BFR regimens.

Besides growth factors, different cytokines have also been demonstrated to influence bone metabolism. One potential candidate that has frequently been discussed is interleukin-6. Previous studies found that interleukin-6 contributes to bone remodeling during early stages of fracture healing (123) and stimulates RANKL production in osteoblastic cells (124). Indeed, levels of interleukin-6 have been reported to be increased after LL-BFR training compared with LL training (120) (Fig. 3). However, direct links to changes in bone remodeling are missing, which limits the ability to draw definitive conclusions at present.

#### Signaling pathways.

Generally, ischemic conditions during LL-BFR training have been found to facilitate metabolic stress (15) and thus lead to physiological elevations of ROS production (125) and increased expression of hypoxia-inducible transcription factors such as hypoxia-inducible factor 1-alpha (HIF-1α) (126) (Fig. 3). Whereas metabolic acidosis per se has been shown to be involved in bone resorption (68), both ROS (127) and HIF-1α (128) have been found to promote osteogenesis. Ha et al. (127) found that ROS act as important mediators of RANKL-induced signaling pathways. As described above, RANKL is essential for the differentiation and activation of osteoclasts and thus bone remodeling. In case of excessive concentrations, ROS have been shown to be involved in apoptosis of osteoblasts (129). Interestingly, previous experiments have highlighted that HIF-1 protects osteoblasts from ROS-induced apoptosis (129) and even plays a critical role in bone formation. Moreover, in bone the HIF pathway has also been shown to independently modulate osteogenic precursor recruitment (130). Within this context, the HIF pathway is involved in upregulating important hypoxia response genes, including vascular endothelial growth factor (VEGF) (131). A tight coupling of osteogenesis and angiogenesis in bone development is crucial, given the important role of vasculature in suppling oxygen, nutrients, and osteoblast and osteoclast precursors for optimal bone regeneration and remodeling (131). Within a recent meta-analysis, Li et al. (126) reported that LL-BFR training elicits more VEGF and HIF-1α mRNA expression compared with low-load exercise without BFR (Fig. 3). This suggests that BFR training might be more conducive to improving vascular function and bone health, but this needs to be investigated in further trials.

### Limitations to Existing Evidence

Although the existing evidence regarding the acute and chronic effect of LL-BFR training on bone metabolism and health is promising, there are several limitations to these data, which may offer an explanation for contrasting findings and also limit the ability to draw accurate conclusions. First, only one study to date has included both of the two key biomarkers of bone metabolism recommended by the National Bone Health Alliance (73), and this study did not examine the acute and/or time course response to an acute bout of LL-BFR exercise training. Furthermore, measurement time points do not necessarily align with the time course response of these biomarkers to acute exercise. For example, CTX-1 has been measured at 30 min (100) and 60 min (101) after exercise in acute LL-BFR studies; however, as demonstrated by Dolan et al. (74) CTX-1 can remain elevated above resting levels at 2 h and even 72 h after exercise, and several hormones relevant to the bone remodeling process are also time sensitive in their response to an acute bout of exercise. It is also important to note that the time course response of key biomarkers of bone metabolism to an acute bout of LL-BFR exercise may differ compared with non-BFR exercise. Therefore, more frequent venous sampling (e.g., via cannulation) may be required to thoroughly investigate the time course response of key biomarkers of bone metabolism to an acute bout of LL-BFR exercise. With regard to study design, only three studies to date (101, 105, 107) have included a nonexercising control group, which is especially important when considering that some biomarkers (e.g., CTX-1) are known to be more influenced by circadian rhythm (83) and levels may therefore naturally change over the course of an experimental protocol independent of an exercise stimulus. Moreover, there is a general lack of control for potentially confounding factors including nutritional status, energy availability, circadian variations, menstrual cycle, and age among studies investigating LL-BFR and bone metabolism and health. Specific to the BFR stimulus itself, there is heterogeneity in methodology of application across the different studies published to date. To enable comparison of results across studies, BFR must be prescribed and applied according to current guidelines of optimal application (132).

### Future Research Directions

Although preliminary evidence highlights the potential effects of LL-BFR training on bone remodeling, heterogeneous study designs and exercise protocols limit generalizable conclusions. Besides the conduct of well-designed, randomized controlled trials, future work should aim for combining several key biomarkers for a global understanding of bone metabolism and associated mechanisms and how this can be manipulated via the induction of local hypoxia. Within the BFR literature, recent guidelines have recommended the use of individualized cuff pressures (i.e., % of the individual’s limb occlusion pressure) (132) since this takes individual anthropometrics and hemodynamics into account, which are often neglected with arbitrary cuff pressure intensities. Unstandardized cuff pressures might ultimately lead to greater variability and heterogeneity in biological responses. Furthermore, alongside correct prescription and application of BFR exercise it is critical that thorough medical screening is conducted before implementation of a BFR exercise intervention, to ensure that any potential risks are mitigated (132). Venous thromboembolism, pulmonary embolism, and rhabdomyolysis are commonly identified as risk factors with BFR exercise (132). The first study to identify these was a large epidemiological study in Japan (133), which reported incidence rates of 0.055% and 0.008% for venous thromboembolism and pulmonary embolism, respectively, in 12,642 individuals within the BFR exercise field. It is important to note that these incidence rates were lower than those in the general population at the time and that a true medical diagnosis for pulmonary embolism was not confirmed. Importantly, several studies have since examined direct blood markers of coagulation (e.g., D-dimer) in response to acute and chronic BFR exercise and reported no changes (134–136). Furthermore, some of these studies were conducted in populations that are often considered to be at a higher risk for venous thromboembolism, such as the elderly and individuals with ischemic heart disease (134, 137). Nevertheless, the majority of this research has been conducted in apparently healthy populations and has not considered variables such as age, sex, and obesity; moreover, certain individuals may be at a heightened risk. With regard to rhabdomyolysis, there is limited evidence, mostly in the form of case reports, that suggests that BFR can cause rhabdomyolysis (138, 139). This has been a source of contention in the literature (140, 141); however, it is generally agreed that the risk of rhabdomyolysis with BFR exercise is primarily driven by inappropriate prescription and application. For example, in the case report by Tabata et al. (139) it is suggested that one acute session of BFR exercise resulted in rhabdomyolysis. What fails to be mentioned is that this individual performed BFR exercise with unnecessarily high pressures, was obese, and had been sedentary for several years and subsequently performed upper and lower limb exercise at higher intensities in the same session. Therefore, the cause of rhabdomyolysis is likely inappropriate prescription of exercise training in general in addition to BFR pressure prescription. However, this emphasizes the importance of correct prescription of BFR exercise parameters, in addition to thorough screening for risk factors and their likelihood. The reader is directed to the key papers by Brandner et al. (142), Nascimento et al. (143), and Patterson et al. (132) for guides on preuse screening and optimal parameters in relation to BFR exercise.

Besides BFR-dependent variables, future studies must control for potential confounding factors that have been suggested to influence the bone metabolic response to exercise. Future work should concentrate on elucidating the precise time course response of specific bone biomarkers and investigate potential physiological mechanisms and strive to investigate causal relationships in terms of LL-BFR and bone health in diverse settings and populations. Since previous experiments by Ashizawa et al. (144) have found that bone metabolism was altered for up to several days after conventional high-load exercise (60–80% 1RM), studies are needed to estimate the effects of low-load exercise combined with BFR. Additionally, there is a lack of studies manipulating different features of specific bone idiosyncrasies including the combined effects of meal and BFR training or dose-response relationship and timing of BFR exercise and bone adaptations. Investigations to obtain knowledge of bone blood perfusion during and as a result of LL-BFR training, and the predominant mechanisms, may contribute toward the development of a novel rehabilitation tool to aid in fracture repair and other bone-related pathologies. Finally, we have developed a methodological framework (Table 2) to facilitate the design of well-controlled, robust studies with relevant biomarkers, to further our understanding of the physiological effect of LL-BFR on bone metabolism and health.

**Table 2.** Methodological framework for investigations on the bone metabolic response to LL-BFR exercise

	Recommended Action	Rationale
Participants	*1*) Include males and females for subgroup analysis	Sex differences in the bone metabolic response to exercise have been reported
	*2*) Control for age and investigate different age groups	Age is known to influence the bone metabolic response to exercise
	*3*) Control for participant activity level	Different habitual activity levels, and types of activity, may influence the bone metabolic response to exercise
	*4*) Control for menstrual cycle phase	This has been shown to impact bone metabolic response to exercise
	*5*) Screen for medical conditions and pharmacological interventions known to influence bone metabolism	These are all potential confounding factors that may influence the bone metabolic response to exercise
	*6*) Control for acute nutritional intake and overall nutritional status	Energy availability and nutritional status may influence the bone metabolic response to exercise
	*7*) Thorough screening for past injuries, particularly fractures, and risk factors related to BFR exercise including venous thromboembolism, pulmonary embolism, and rhabdomyolysis	History of a previous fracture may influence the bone metabolic response to exercise
Study design	*1*) Inclusion of a nonexercising control group, with matched measurement time points	To ensure that changes in bone modeling biomarkers directly relate to the intervention itself, and not other factors (e.g., circadian variation)
	*2*) Control for time of day	Specific bone modeling biomarkers are more sensitive to circadian variations than others
BFR protocol	*1*) Applied pressure should be individualized relative to limb occlusion pressure	This is the current gold standard of BFR pressure prescription.
	*2*) Employ a range of pressures at different percentages of limb occlusion pressure	Existing data indicate that the level of pressure applied may influence the acute physiological response and chronic adaptation to LL-BFR
	*3*) Employ LL-BFR exercise according to current guidelines for optimal application	To ensure LL-BFR exercise is applied correctly
Outcome measures	*1*) Include, as a minimum, the biomarkers CTX-1 and P1NP to assess bone resorption and formation processes, as per National Bone Health Alliance Recommendations	Higher specificity to bone metabolism and smaller biological variability compared with other markers
	*2*) Include assessment of PTH and calcitonin levels	To investigate calcium metabolism
	*3*) For acute studies, examine the time course response of each biomarker, according to the known time course response of each, and include additional time points	Existing data demonstrate that peak changes in bone modeling biomarkers in response to exercise occur at different time points for different biomarkers
	*4*) For chronic studies, include objective measures of bone microarchitecture (e.g., high-resolution peripheral quantitative computed tomography or magnetic resonance imaging) and bone mineral density (e.g., dual-energy X-ray absorptiometry)	Objective and quantifiable measurements of bone health

BFR, blood flow restriction; CTX-1, COOH-terminal telopeptide of type I collagen; LL-BFR, low-load exercise with blood flow restriction; P1NP, NH_2_-terminal propeptide of type 1 procollagen; PTH, parathyroid hormone.

## DISCLOSURES

No conflicts of interest, financial or otherwise, are declared by the authors.

## AUTHOR CONTRIBUTIONS

L.H. and C.C. conceived and designed research; L.H. and C.C. prepared figures; L.H. and C.C. drafted manuscript; L.H. and C.C. edited and revised manuscript; L.H. and C.C. approved final version of manuscript.
